# Predictors of Decannulation Success in Tracheostomy: A 10‐Year Analysis of the Global Tracheostomy Collaborative Database

**DOI:** 10.1002/ohn.70013

**Published:** 2025-09-02

**Authors:** Charissa J. Zaga, Carly E. Milliren, Brendan A. McGrath, Christina J. Yang, Bradley A. Schiff, Stephen J. Warrillow, Jennifer K. Henningfeld, Prudence A. Gregson, Joshua R. Bedwell, Karen M. Beaudet, Michael J. Brenner, Vinciya Pandian

**Affiliations:** ^1^ Department of Speech Pathology Division of Allied Health, Austin Health Melbourne Australia; ^2^ Implementation Science Unit, Institute for Breathing and Sleep Austin Health Melbourne Australia; ^3^ Department of Critical Care University of Melbourne Melbourne Australia; ^4^ Department of Audiology and Speech Pathology University of Melbourne Melbourne Australia; ^5^ Biostatistics and Research Design Center Boston Children's Hospital Boston Massachusetts USA; ^6^ Data Core Team Global Tracheostomy Collaborative Chicago Illinois USA; ^7^ NHS Foundation Trust, National Tracheostomy Safety Project University of Manchester Manchester UK; ^8^ Department of Otorhinolaryngology–Head and Neck Surgery, Montefiore Medical Center Albert Einstein College of Medicine Bronx New York USA; ^9^ Austin Health Melbourne Australia; ^10^ Tracheostomy and Home Ventilator Program, Division of Pulmonology, Department of Pediatrics Children's Wisconsin Milwaukee Wisconsin USA; ^11^ The Tracheostomy Review and Management Service Austin Health Melbourne Australia; ^12^ Department of Otolaryngology Baylor College of Medicine Houston Texas USA; ^13^ Texas Children's Center Houston Texas USA; ^14^ Pediatric Otolaryngology Children's Mercy Kansas City Missouri USA; ^15^ Department of Otolaryngology–Head & Neck Surgery University of Michigan Medical School Ann Arbor Michigan USA; ^16^ Otolaryngology–Head and Neck Surgery College of Medicine Hershey Pennsylvania USA; ^17^ Immersive Learning and Digital Innovations, Ross and Carol Nese College of Nursing The Pennsylvania State University University Park Pennsylvania USA

**Keywords:** adverse events, airway, clinical outcomes, clinical practice guidelines, critical care, decannulation, hospital utilization, ICU admission, intensive care unit, laryngotracheal injury, length of stay, mechanical ventilation, mortality, multidisciplinary care, patient safety, patient‐centered care, quality improvement, quality of life, rehabilitation, reintubation, risk factors, speech, subglottic stenosis, survival, survivorship, swallowing, tracheostomy, tracheotomy, ventilator, ventilatory support

## Abstract

**Objective:**

Decannulation is a critical milestone in functional recovery after tracheostomy, but standardized guidelines are lacking. This study examined factors associated with tracheostomy decannulation success, comparing hospital utilization, adverse events, and survival outcomes between decannulated and non‐decannulated patients.

**Study Design:**

Retrospective, observational study.

**Setting:**

Data were collected from 25 hospitals participating in the Global Tracheostomy Collaborative (GTC) in the United States, Australia, and the United Kingdom.

**Methods:**

Prospectively collected data from adult patients who underwent tracheostomy from 2013 to 2022 were analyzed. Outcomes included decannulation success, hospital utilization metrics (intensive care unit [ICU] admissions, mechanical ventilation use, tracheostomy duration, and hospital length of stay), survival to discharge, discharge destinations, and adverse events. Associations were tested using *t* tests, chi‐square, and Fine‐Gray models, adjusting for clustering by site.

**Results:**

Among 5318 patients, 52.9% were decannulated before discharge. Predictors of decannulation included younger age, fewer comorbidities, elective and surgical admissions, and upper airway obstruction as an indication for tracheostomy versus facilitation of ventilation (all *P* < .001). Geographic variations were significant, with higher decannulation rates in Australia (82.1%) and the United Kingdom (70%) compared to the United States (13.5%) (*P* < .001). Decannulated patients had not only higher survival rates but also higher adverse events (11.4%, *P* = .002), particularly unplanned decannulation. Discharge destination varied by country, with the United Kingdom having the highest home discharge rate (*P* < .001).

**Conclusion:**

Decannulation success is associated with patient and institutional factors, suggesting the need for standardized protocols to promote equitable tracheostomy management. Geographic variations in decannulation rates, adverse events, and hospital utilization suggest opportunities for harmonized guidelines to enhance outcomes and resource allocation.

Tracheostomy is a common airway access procedure, but practices around aftercare and decannulation vary based on patient, provider, and contextual factors.^1^, ^2^, ^3^, ^4^ Tracheostomy may be performed for indications including need for prolonged mechanical ventilation, upper airway obstruction, airway protection, or pulmonary hygiene.^1^, ^5^, ^6^, ^7^, ^8^, ^9^, ^10^, ^11^ The incidence of tracheostomy varies geographically due to differences in healthcare systems, patient populations, and clinical practices. In resource‐rich countries, approximately 250,000 tracheostomies are performed annually,^1^ with approximately 100,000 in the United States.^12^ In the United Kingdom, approximately 15,000 tracheostomies are performed each year,^13^ whereas 20,000 are performed annually in Australia and New Zealand.^10^ Although tracheostomy can facilitate ventilator weaning and improve patient outcomes in critical care,^14^, ^15^, ^16^, ^17^ it is associated with significant periprocedural and post‐procedural complications such as tube displacement, tube obstruction, bleeding, infection, tracheal stenosis, and granulation tissue formation, associated with morbidity and mortality.^11^, ^14^, ^15^, ^16^, ^17^, ^18^, ^19^, ^20^, ^21^, ^22^, ^23^, ^24^ Therefore, tracheostomy decannulation is a key goal, performed once a patient can breathe independently, manage secretions, and protect the airway.^25^, ^26^, ^27^, ^28^ This assessment often requires interprofessional collaboration for optimal patient‐centered care.^28^, ^29^, ^30^, ^31^, ^32^

Patients and families commonly report negative experiences related to tracheostomy, including reduced well‐being and quality of life, difficulties caring for the tracheostomy, changes to speech and communication, disfigurement and altered body image, stigma, and social withdrawal.^33^, ^34^, ^35^, ^36^ From a patient's perspective, decannulation represents a major milestone toward independence and a return to a more normal way of life. The ability to breathe freely, speak without assistance, and eat and drink normally can enhance physical and psychosocial well‐being.^35^, ^37^ Healthcare professionals strive for safe and efficient tracheostomy decannulation, but the decision‐making process is complex, influenced by numerous factors affecting both timing and outcomes.^38^, ^39^ However, decannulation decision‐making is complex, influenced by patient and institutional factors, including variability in clinical protocols. From a healthcare system perspective, prolonged tracheostomy care imposes substantial burdens, particularly regarding staff training requirements and the need for high‐acuity care settings.^13^

There are no universally standardized criteria for its initiation, leading to practice variability, delayed or failed decannulation, emergency tracheostomy tube reinsertion or reintubation, readmissions to the intensive care unit (ICU)/hospital, and other complications.^40^, ^41^, ^42^, ^43^, ^44^, ^45^, ^46^, ^47^, ^48^ Moreover, limited multicenter data exist regarding the characteristics and hospital utilization patterns of tracheostomy patients and their outcomes post‐decannulation, creating gaps in evidence‐based practice. Whereas most studies investigating decannulation outcomes have been single‐institution studies, retrospective analysis, or rely on administrative data, this study leverages data from a large, prospectively collected international registry on tracheostomy procedures and care. This study aims to identify factors influencing decannulation success by examining demographic and clinical characteristics, comorbidities, and hospital utilization metrics. Additionally, it assesses survival outcomes between decannulated and non‐decannulated patients, analyzes geographic variations, and identifies factors associated with the timing of decannulation and adverse events.

## Methods

### Study Design

This retrospective observational study utilized anonymized data from the Global Tracheostomy Collaborative (GTC) database, which includes prospectively collected tracheostomy care data from 56 hospitals worldwide. The GTC database captures data from both primary admissions (when the tracheostomy tube is inserted during that admission) and secondary admissions (when the patient is admitted with a tracheostomy tube in situ). The GTC database is a HIPAA‐compliant database that member hospitals enter anonymous patient‐level metrics into the REDCap web platform hosted by Vanderbilt University.^49^ Ethics approval was obtained from the University of Michigan Institutional Review Board (IRB # HUM00208783), and the study was reported in accordance with the Strengthening the Reporting of Observational Studies in Epidemiology (STROBE) statement (Supplemental Table S1, available online).^50^

### Study Population and Inclusion Criteria

The inclusion criteria focused on adult patients aged 18 years and older who underwent a tracheostomy placement during a primary hospital admission. Data were included from hospitals that contributed at least 10 eligible patients during the study period from 2013 to 2022. This timeframe was selected to ensure a comprehensive analysis of tracheostomy care and outcomes over a decade, and data reliability. Patients were excluded if data on decannulation status, tracheostomy duration, or survival status at discharge were incomplete or missing to ensure reliable analysis of predictors and outcomes.

### Measures

Data obtained from the GTC database included patient demographics (age, sex, comorbidities, and country) and clinical details such as the indication for tracheostomy, admission type (emergency or planned), and admission reason (medical or surgical). The primary outcome was decannulation status. Patients were categorized based on whether they were successfully decannulated before hospital discharge (decannulated group) or retained their tracheostomy at discharge (non‐decannulated group). Secondary outcomes included hospital utilization, survival, and adverse events. Hospital utilization metrics included ICU admission, mechanical ventilation use, tracheostomy duration, and hospital length of stay. Survival outcomes included hospital mortality and discharge destination. Adverse events were events that occurred during hospitalization before decannulation, including unplanned (accidental) decannulation, tracheostomy‐related bleeding, tube obstruction (partial or complete), and failed decannulation attempts. For patients who were ultimately decannulated, these adverse events were recorded before successful decannulation, not as post‐decannulation complications.

### Statistical Analysis

Descriptive statistics include mean (standard deviation) or median (interquartile range; IQR) for continuous variables, and frequency (percent) for categorical variables. Associations between decannulated and non‐decannulated patients were examined using two‐sample *t* tests or Wilcoxon rank‐sum tests for continuous variables, and chi‐square tests for categorical variables. Variation by country in the distributions of patient characteristics, hospital utilization, and decannulation was examined using chi‐square tests for categorical variables and *t* tests or Wilcoxon rank‐sum tests for continuous variables. In addition, similar analyses were performed stratified by country to examine associations between decannulation status and patient characteristics within each country. Competing events survival regression analysis (Fine‐Gray hazard model) was used to examine the adjusted associations between time‐to‐successful decannulation and demographic and clinical factors, treating death before decannulation as a competing risk. Patients who survived to discharge but were not decannulated were censored at the time of discharge. A robust sandwich estimator was used to adjust for clustering by hospital sites. Analyses were conducted using SAS Software (version 9.4) with an alpha level set at .05.^51^

## Results

### Selection of Eligible Patients From the GTC Database

**Figure 1:**
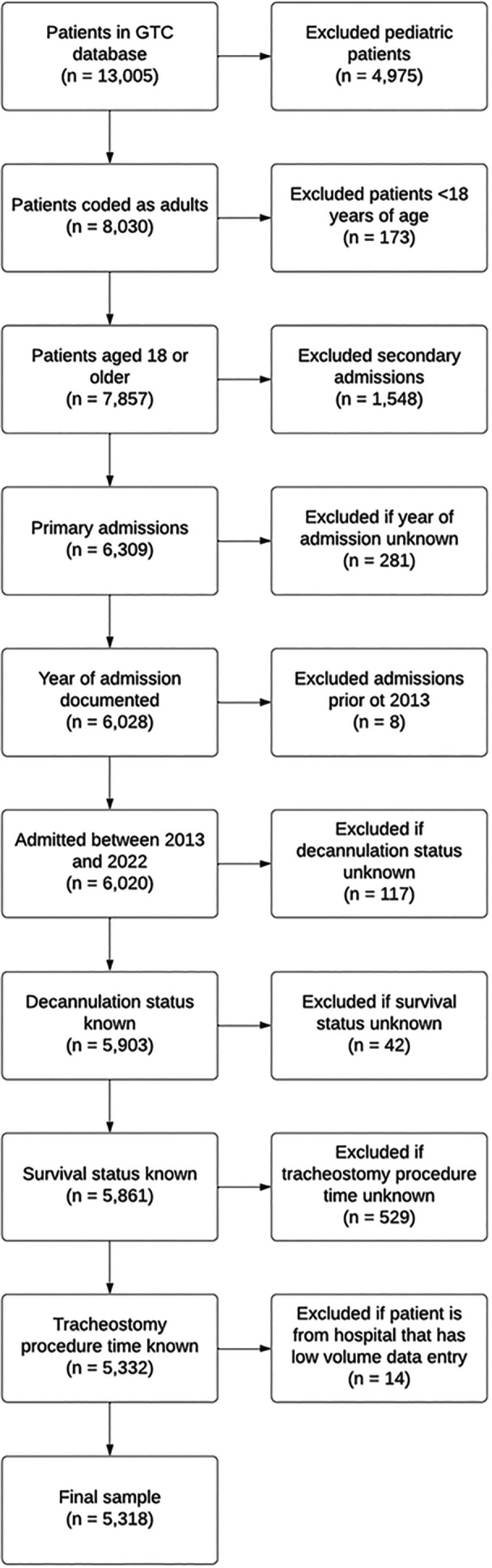
Flow chart for selection of sample for analysis. Figure depicts sequential hierarchical steps taken for selection of patient cases meeting criteria for inclusion in patient‐level data analysis.

The GTC database includes information from 13,005 patients across 56 hospitals. After applying eligibility criteria and relevant exclusions, an initial sample of 5332 patients from 32 hospitals was identified. Further refinement based on data robustness and hospital engagement criteria yielded a final cohort of 5318 adult patients from 25 hospitals, including 15 in the United Kingdom, 6 in Australia, and 4 in the United States, which were included in the analysis (Figure 1). The geographic distribution was as follows: United Kingdom, 1971 patients (37.1%); United States, 1917 patients (36.1%); and Australia, 1430 patients (26.9%).

### Demographic and Clinical Characteristics and Comorbidities

#### Demographic Characteristics

The overall mean age was 57.9 years (SD = 15.4), with the US cohort being older (59.1 years, SD = 15.0) compared to the United Kingdom (58.4 years, SD = 14.6) and Australia (55.5 years, SD = 16.8) (*P* < .001). Males comprised 63.7% of the total cohort, with the highest proportion in Australia (66.3%) and the lowest in the United States (59.4%) (*P* < .001) (Supplemental Table S2, available online). Of the 5318 patients, 2813 (52.9%) were successfully decannulated before discharge. The mean age of decannulated patients was 56.2 years (SD = 15.6), significantly younger than non‐decannulated patients, with a mean age of 59.8 years (SD = 15.0) (*P* < .001). Younger age categories were associated with a higher likelihood of decannulation (*P* < .001). A higher proportion of males versus females were decannulated by discharge (54.6% of males vs 49.9% of females; *P* = .001). The primary indication for tracheostomy differed across regions (*P* < .001). Facilitation of ventilation was the most common reason overall (69.7%), though it was significantly more frequent in the United States (76.1%) than in the United Kingdom (71.7%) or Australia (58.5%) based on analysis of indication of facilitation of ventilation by country (United Kingdom vs United States *P* = .002; United Kingdom vs Australia *P* < .001; United States vs Australia *P* < .001). In contrast, upper airway obstruction was a more frequent indication in Australia (33.9%) compared to the United Kingdom (23.1%) and the United States (19.1%) (*P* < .001) (Supplemental Table S3 and Figure S1, available online). Primary admission diagnoses varied significantly (*P* < .001) across countries, with neurological conditions (34.6%) most common in the United Kingdom (38.9%), respiratory failure (41.2%) highest in the United States (44.7%), and head and neck pathology (24.2%) most prevalent in Australia (29.1%). Decannulation varied by country with Australia having the highest proportion of patients decannulated by discharge (82.1%), followed by the United Kingdom (70.0%). The United States had a significantly higher proportion of patients not decannulated by discharge (86.5%) (*P* < .001) (see Table 1).

**Table 1:** Demographic and Clinical Characteristics and Comorbidities for Patients Who Were Decannulated Versus Not Decannulated by Hospital Discharge (N = 5318)

Patient characteristics	n (%)		*P*‐value
	Decannulated by discharge		
	Yes (n = 2813)	No (n = 2505)	
*Demographic characteristics*			
Age, y, mean (SD)	56.2 (15.6)	59.8 (15.0)	<.001
Age category			<.001
<20 y	29 (1.0%)	13 (0.5%)	
20‐29 y	183 (6.5%)	112 (4.5%)	
30‐39 y	239 (8.5%)	165 (6.6%)	
40‐49 y	406 (14.4%)	263 (10.5%)	
50‐59 y	657 (23.4%)	559 (22.3%)	
60‐69 y	728 (25.9%)	706 (28.2%)	
70 y or older	571 (20.3%)	687 (27.4%)	
Male sex	1848 (65.7%)	1537 (61.4%)	.001
Country			<.001
United Kingdom	1380 (49.1%)	591 (23.6%)	
United States	259 (9.2%)	1658 (66.2%)	
Australia	1174 (41.7%)	256 (10.2%)	
*Clinical characteristics*			
Reason for tracheostomy			<.001
Facilitation of ventilation	1819 (64.7%)	1888 (75.4%)	
Upper airway obstruction	841 (29.9%)	465 (18.6%)	
Aspiration	58 (2.1%)	79 (3.2%)	
Secretion retention	19 (0.7%)	16 (0.6%)	
Other	69 (2.5%)	48 (1.9%)	
Unknown	7 (0.3%)	9 (0.4%)	
Scheduled admission			<.001
Emergent	1713 (60.9%)	2068 (82.6%)	
Scheduled	1084 (38.5%)	431 (17.2%)	
Unknown	16 (0.6%)	6 (0.2%)	
Reason for admission			<.001
Medical	1263 (44.9%)	1752 (69.9%)	
Surgical	1549 (55.1%)	751 (30.0%)	
Unknown	1 (<0.1%)	2 (0.1%)	
*Comorbid systems (not mutually exclusive)*			
Cardiovascular	1295 (46.0%)	1645 (65.7%)	<.001
Respiratory	876 (31.1%)	1413 (56.4%)	<.001
Metabolic/endocrine/genetic	554 (20.0%)	865 (34.5%)	<.001
Neurological	394 (14.0%)	844 (33.7%)	<.001
Gastrointestinal	498 (17.7%)	707 (28.2%)	<.001
Renal	316 (11.2%)	621 (24.8%)	<.001
Oncological	565 (20.1%)	362 (14.5%)	<.001
Hematological/immunological	192 ((6.8%)	474 (18.9%)	<.001
Sepsis	201 (7.2%)	434 (17.3%)	<.001
Musculoskeletal/skin	218 (7.8%)	320 (12.8%)	<.001
Trauma/injury	94 (3.3%)	78 (3.1%)	.64
Other	472 (16.8%)	405 (16.2%)	.55
Number of comorbid organ systems			<.001
None	358 (12.7%)	140 (5.6%)	
1	834 (29.7%)	392 (15.7%)	
2	656 (23.3%)	499 (19.9%)	
3	461 (16.4%)	369 (14.7%)	
4	240 (8.5%)	357 (14.3%)	
5 or more	219 (7.8%)	710 (28.3%)	
Unknown	45 (1.6%)	38 (1.5%)	

#### Clinical Characteristics

Most patients received a tracheostomy for the facilitation of ventilation regardless of whether they were decannulated (see Table 1). Upper airway obstruction was the second most common reason among decannulated patients (29.9%) compared to 18.6% in non‐decannulated patients (*P* < .001). The type of admission, emergent or scheduled, and medical or surgical also correlated with decannulation outcomes (*P* < .001). Decannulation occurred in 45.3% of emergent admissions compared to 71.6% among scheduled admissions. The reason for admission was associated with decannulation status (*P* < .001). Surgical admissions were more prevalent among decannulated patients (55.1% vs 30.0%), whereas medical reasons for admission were more common among non‐decannulated patients (69.9% vs 44.9%) (see Table 1).

#### Comorbidities

Cardiovascular, respiratory, metabolic/endocrine/genetic, neurological, gastrointestinal, renal, oncological, hematological/immunological, sepsis, and musculoskeletal/skin conditions comorbidities were significantly more common in non‐decannulated patients (Table 1). In contrast, trauma/injury and other unspecified comorbidities did not differ significantly in prevalence between the groups. The number of comorbid organ systems was also associated with decannulation status (*P* < .001) with a higher proportion of decannulated patients having no comorbidities (12.7% vs 5.6%) or only one comorbid organ system (29.7% vs 15.7%) compared to non‐decannulated patients. Non‐decannulated patients were more likely to have five or more comorbid organ systems (28.3% vs 7.8%).

### Hospital Utilization Metrics, Survival to Discharge, and Discharge Destinations

#### Hospital Utilization

In the overall cohort, most patients in both groups were admitted to the ICU, with a slightly higher proportion observed among those decannulated by discharge (97.6% vs 93.6%, *P* < .001) (Table 2). The median tracheostomy duration by discharge was comparable between the groups (16 days for decannulated patients vs 15 days for non‐decannulated patients). However, the length of hospital stay was significantly longer for decannulated patients, with a median of 43 days (IQR = 45.0), compared to 32 days (IQR = 31.0) for those not decannulated (*P* < .001). The median length of hospital stay was longest in the United Kingdom (44 days, IQR = 47.0), followed by Australia (41 days, IQR = 40.0), and shortest in the United States (28 days, IQR = 28.0) (*P* < .001). Similarly, median tracheostomy duration was longest in Australia (19 days, IQR = 22.0), followed by the United Kingdom (17 days, IQR = 24.0), and shortest in the United States (13 days, IQR = 15.0) (*P* < .001). Survival to discharge was highest in Australia (89.0%), followed by the United States (87.8%), and lowest in the United Kingdom (84.4%) (*P* < .001). Discharge home was most common in the United Kingdom (62.0%), whereas 40.9% of US patients were discharged to long‐term care facilities (*P* < .001), highlighting regional differences in post‐acute tracheostomy care pathways.

**Table 2:** Hospital Utilization and Survival for Patients Who Were Decannulated Versus Not Decannulated by Hospital Discharge (N = 5318)

Patient characteristic	n (%)		*P*‐value
	Decannulated by discharge		
	Yes (n = 2813)	No (n = 2505)	
*Hospital utilization and survival*			
Any admission to ICU	2746 (97.6%)	2344 (93.6%)	<.001
Any invasive mechanical ventilation	2508 (89.2%)	2211 (88.3%)	.25
Tracheostomy time, d, median (IQR)	16.0 (19.0)	15.0 (21.0)	.28
Length of stay, d, median (IQR)	43.0 (45.0)	32.0 (31.0)	<.001
Survival to discharge	2667 (94.8%)	1952 (77.9%)	<.001
Discharge destination (among those alive at d/c)			<.001
Home	1573 (59.0%)	389 (19.9%)	
Rehabilitation hospital	770 (28.9%)	256 (13.1%)	
Long‐term care facility	35 (1.3%)	712 (36.5%)	
Acute care hospital	204 (7.7%)	256 (13.1%)	
Skilled nursing facility	56 (2.1%)	259 (13.3%)	
Other	18 (0.7%)	72 (3.7%)	
Unknown	11 (0.4%)	8 (0.4%)	

Abbreviations: ICU, intensive care unit; IQR, interquartile range.

#### Survival to Discharge and Discharge Destinations

In the whole cohort analysis, survival to discharge differed by decannulation status, with 94.8% of decannulated patients surviving to discharge compared to 77.9% of non‐decannulated patients (*P* < .001). Among those who were alive at discharge, a majority of decannulated patients were discharged home (59.0%), contrasted with only 19.9% of non‐decannulated patients (Table 2). Rehabilitation hospitals were the next common destination for decannulated patients (28.9%), while a substantial proportion of non‐decannulated patients were transferred to long‐term care facilities (36.5%).

### Decannulation, Hospital Utilization, Survival, and Discharge Destinations by Geographical Location

Australia had the highest proportion of patients decannulated by primary hospital discharge (82.1%), followed by the United Kingdom (70.0%) and the United States (13.5%), with the median tracheostomy time being longest in Australia (19 days) and shortest in the United States (13 days) (Table 3). The median length of hospital stay was longest in the United Kingdom (44 days) and Australia (41 days) and shortest in the United States (28 days) (*P* < .001). Survival to discharge was highest in Australia (89.0%), with the United States slightly lower (87.8%) and the lowest in the United Kingdom (84.4%) (*P* < .001). Discharge destinations varied significantly (*P* < .001), with a higher proportion of patients in the United Kingdom being discharged home (62.0%) compared to the United States (23.2%) and Australia (42.4%), whereas the United States had a notably higher proportion of discharges to long‐term care facilities (40.9%).

**Table 3:** Hospital Utilization, Survival, and Discharge Destination by Country of Site From 25 Hospitals Participating in the Global Tracheostomy Collaborative (N = 5318)

Patient characteristic	n (%)			*P*‐value
	United Kingdom (n = 1971)	United States (n = 1917)	Australia (n = 1430)	
Decannulated by discharge	1380 (70.0%)	259 (13.5%)	1174 (82.1%)	<.001
Tracheostomy duration, d, median (IQR)	17.0 (24.0)	13.0 (15.0)	19.0 (22.0)	<.001
Length of stay, d, median (IQR)	44.0 (47.0)	28.0 (28.0)	41.0 (40.0)	<.001
Survival to discharge	1664 (84.4%)	1683 (87.8%)	1272 (89.0%)	<.001
Discharge destination^a^				<.001
Home	1032 (62.0%)	391 (23.2%)	539 (42.4%)	
Rehabilitation hospital	285 (17.1%)	202 (12.0%)	539 (42.4%)	
Long‐term care facility	42 (2.5%)	689 (40.9%)	16 (1.3%)	
Acute care hospital	279 (16.8%)	27 (1.6%)	154 (12.1%)	
Skilled nursing facility	14 (0.8%)	294 (17.5%)	7 (0.6%)	
Other	6 (0.4%)	70 (4.2%)	14 (1.1%)	
Unknown	6 (0.4%)	10 (0.6%)	3 (0.2%)	

Abbreviation: IQR, interquartile range.

^a^ Among those alive at discharge.

### Adverse Events Rates Per 1000 Tracheostomy‐Bed Days

Just over a tenth of patients (11.4%) decannulated by discharge experienced adverse events before decannulation, a rate significantly higher than the 8.6% observed in patients not decannulated (*P* < .001). Adverse events included unplanned (accidental) decannulation, tracheostomy‐related bleeding, tube obstruction (partial or complete), one‐way valve placed with an inflated cuff, and failed decannulation (attempt). Unplanned decannulation was more prevalent among the decannulated group (5.2%) compared to the non‐decannulated by discharge group (2.3%) (*P* < .001). Tracheostomy‐related bleeding rates were similar between the decannulated (3.1%) and non‐decannulated groups (4.0%, *P* = .06), as were tube obstruction risks (1.5% vs 1.4%, *P* = .68). Additionally, the incidence of using a one‐way valve with an inflated cuff showed no difference between groups (0.7% in the decannulated group vs 0.9% in the non‐decannulated group, *P* = .49). Failed decannulation was more frequent among those successfully decannulated (1.8%) versus those who were not decannulated by discharge (0.9%, *P* = .004) (Table 4). The rate of unplanned decannulation was highest in Australia (3.4%), followed by the United Kingdom (1.7%) and the United States (1.8%) (*P* = .014). Tracheostomy‐related bleeding was slightly higher in the United States (4.0%) compared to the United Kingdom (2.0%) and Australia (2.4%) (*P* = .06).

**Table 4:** Adverse Events for Patients Who Were Decannulated Versus Not Decannulated by Hospital Discharge (N = 5318)

Adverse events	Decannulated by discharge n (%)		*P*‐value
	Yes (n = 2813)	No (n = 2505)	
Any AE reported	320 (11.4%)	216 (8.6%)	<.001
Unplanned decannulation	146 (5.2%)	58 (2.3%)	<.001
Tracheostomy‐related bleeding	87 (3.1%)	101 (4.0%)	.06
Tube obstruction	42 (1.5%)	34 (1.4%)	.68
One‐way valve placed with cuff inflated	20 (0.7%)	22 (0.9%)	.49
Failed decannulation	51 (1.8%)	22 (0.9%)	.004

Abbreviation: AE, adverse events.

### Factors Associated With Time‐to‐Successful Decannulation

**Figure 2:**
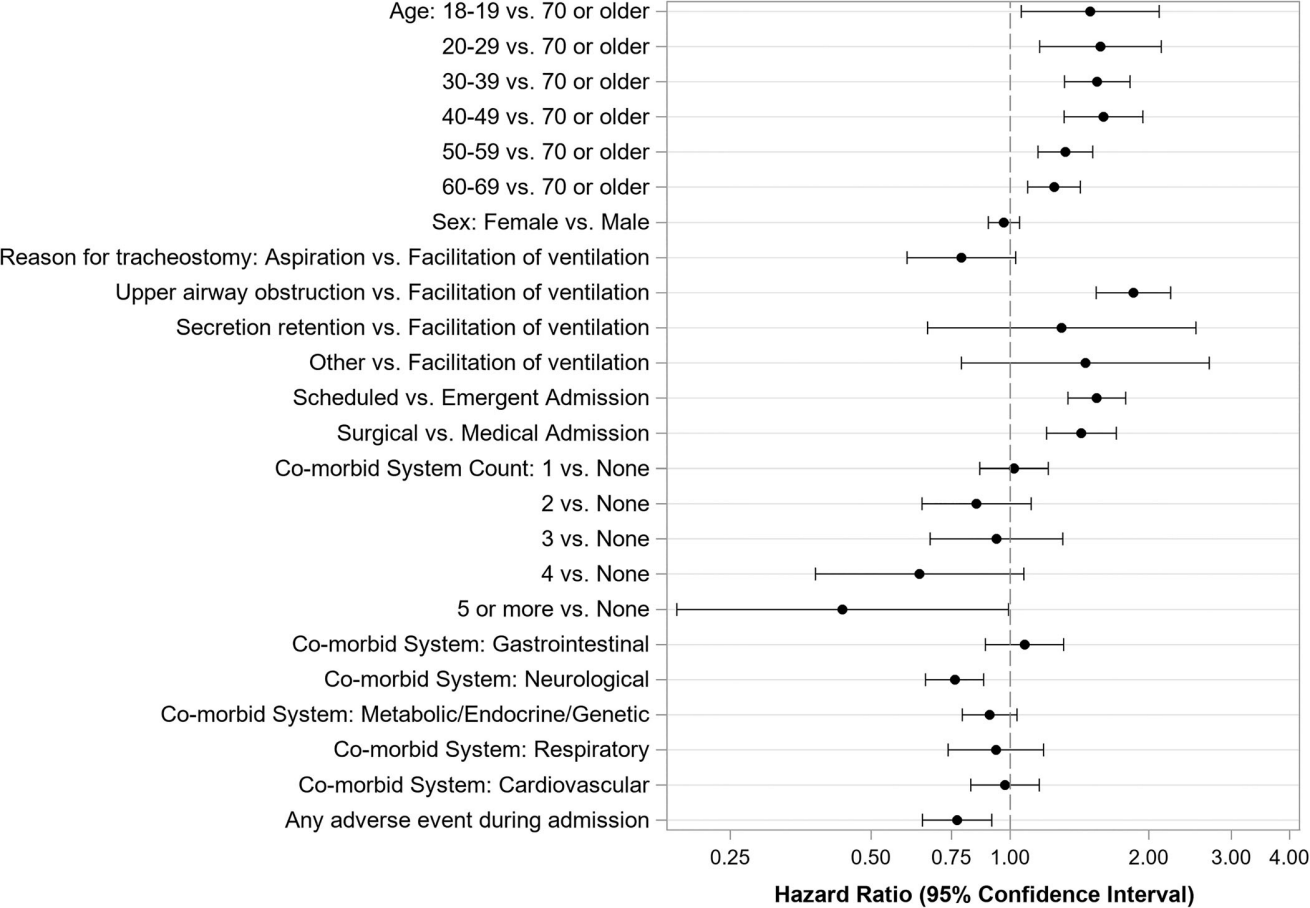
Predictors of decannulation outcome: hazard ratios with confidence interval for decannulation success are depicted for attributes including age, reason for tracheostomy, admission type, comorbid conditions, and adverse events. GTC, Global Tracheostomy Collaborative.

Results from an adjusted regression model examining time‐to‐decannulation showed that younger age was associated with a higher likelihood of successful decannulation, with each lower age decade being more likely to be decannulated compared to those aged 70 or older (Figure 2). Adjusting for other demographic and clinical factors, there was no difference in the likelihood of decannulation by sex (hazard ratio [HR] = 0.97, female vs male; 95% CI: 0.90‐1.05; *P* = .45). Patients with a tracheostomy for upper airway obstruction were more likely to be decannulated compared to those with a tracheostomy for ventilation facilitation (HR = 1.85; 95% CI: 1.54‐2.22; *P* < .001). No significant differences were observed for other tracheostomy indications, including aspiration (HR = 0.79; 95% CI: 0.60‐1.03; *P* = .08), secretion retention (HR = 1.29; 95% CI: 0.66‐2.52; *P* = .45), or any other indication (HR = 1.45; 95% CI: 0.79‐2.69; *P* = .23).

Additional findings were noted relating to elective status and comorbidities. Patients with scheduled admissions were more likely to be decannulated compared to emergent admissions (HR = 1.54; 95% CI: 1.33‐1.78; *P* < .001), and surgical admissions had a higher likelihood of decannulation compared to medical admissions (HR = 1.43; 95% CI: 1.20‐1.70; *P* < .001). The likelihood of decannulation decreased as the number of comorbidities increased, although the only difference that reached statistical significance was 5 or more comorbidities versus none (HR = 0.44; 95% CI: 0.19‐0.99; *P* = .048). Of the specific comorbid systems, only neurological conditions were associated with a lower likelihood of decannulation (HR = 0.76; 95% CI: 0.66‐0.88; *P* < .001). In the adjusted model, experiencing a tracheostomy‐related adverse event during admission was associated with a lower likelihood of successful decannulation (HR = 0.77; 95% CI: 0.65‐0.91; *P* = .003).

## Discussion

This study provides a multi‐institutional perspective on tracheostomy decannulation outcomes by comparing 5318 patients across healthcare systems in the United Kingdom, the United States, and Australia. Although previous studies have assessed tracheostomy decannulation in single‐center or single‐country cohorts, this analysis is among the largest to evaluate decannulation outcomes using prospectively collected, multi‐institutional data. A key finding is the significant variation in decannulation rates across the three regions, with Australia (82.1%) and the United Kingdom (70.0%) demonstrating substantially higher decannulation success compared to the United States (13.5%).

In the analysis of hospital length of stay between countries, we found notable differences between US centers versus the United Kingdom and Australia. The country‐specific patterns in length of stay and discharge destination are consistent with prior data from Australia,^47^ the United Kingdom,^13^ and the United States.^52^ These findings likely reflect structural differences in healthcare systems. Discharge home for UK patients is higher than previously reported,^53^ potentially reflecting increased use of “hospital at home,” models, allowing hospital or ICU‐level care to be delivered in a patient's home.

This approach integrates high‐acuity treatments such as intravenous medications, oxygen therapy, and continuous monitoring along with telehealth and wearable device monitoring. The “Hospital at Home” model saw earlier adoption in the United Kingdom and Australia to alleviate hospital demand, whereas in the United States, its expansion was catalyzed by the COVID‐19 pandemic, which accelerated regulatory support and reimbursement options for home‐based acute care. The hospital discharge data are consistent with previously published Australian data.^29^ Hospital survival of 13% for this cohort compares favorably with previous studies reporting mortality of 13% to 19%.^47^, ^53^, ^54^, ^55^, ^56^

The GTC has collaborated with more than 70 hospitals across multiple countries, including in‐depth studies on the patient journey.^1^ In the United Kingdom, patients with new tracheostomies are typically managed within a single hospital system for their entire inpatient stay. This “one‐stop” approach likely contributes to a higher rate of primary hospital decannulation compared to countries where patients are often transferred to another facility for ongoing rehabilitation with the tracheostomy still in place. When tracheostomies are performed to assist with ventilator weaning in critically ill patients, a higher mortality rate is anticipated. Therefore, death with a tracheostomy still in place leads to a shorter hospital length of stay and a classification of non‐decannulated status, given that many do not recover enough to attempt decannulation. Further data collection is necessary to understand the implications of these differences in healthcare systems, particularly how variations in tracheostomy management pathways impact decannulation success, hospital resource utilization, and long‐term patient outcomes.

In line with existing literature, patient characteristics were an important predictor of successful decannulation. Younger patients had a higher likelihood of decannulation than those aged 70 or older,^38^, ^39^ whereas there were no significant differences in decannulation success based on sex. The reason for tracheostomy placement significantly influenced decannulation outcomes. Patients with tracheostomies placed for upper airway obstruction had the highest likelihood of successful decannulation compared to those requiring tracheostomy primarily for ventilatory support.

Although previous studies suggested that tracheostomy placed for aspiration may reduce the likelihood of decannulation,^54^ our findings did not reach statistical significance. This suggests that while aspiration may be a clinical concern, its direct impact on decannulation success remains uncertain and warrants further investigation in larger, more targeted cohorts. The reason for admission was related to outcomes as well; patients admitted electively or for surgical reasons had a higher decannulation success rate, consistent with prior work.^39^ Additionally, comorbidities significantly influenced decannulation, with a greater number of underlying conditions, particularly neurological disorders, correlating with reduced success.^39^ These results highlight the complexity of tracheostomy care and the importance of individualized patient assessment. Further research could deepen our understanding of these interactions, refining clinical strategies to improve tracheostomy outcomes.

### Study Limitations

This study has several limitations. The observational design and registry‐based data preclude causal inferences about patient characteristics, hospital metrics, and decannulation outcomes. The GTC database primarily includes hospitals with structured tracheostomy programs, potentially limiting generalizability to institutions with less standardized care or those in resource‐limited settings. Selection bias may also exist, as only data from actively participating hospitals were analyzed, and patients with incomplete records were excluded. Further discussion of differences between countries and study limitations appears in Supplemental Appendix 1, available online.

## Conclusion

This study presents comprehensive, comparative evidence of adult tracheostomy decannulation patterns across three major healthcare systems, identifying significant practice variations that impact patient outcomes. While younger age, fewer comorbidities, and elective admissions were associated with higher decannulation success, geographic differences in care pathways and institutional protocols also played a critical role in decannulation rates, length of hospital stay, and discharge planning. The observed disparities in decannulation practices underscore the need for a standardized, internationally recognized protocol to ensure equitable, high‐quality tracheostomy care. These findings provide a strong foundation for developing evidence‐based guidelines that can be implemented through the GTC and other healthcare networks. Future research should focus on protocol harmonization efforts, interventions to improve decannulation rates in low‐performing regions, and strategies to enhance interdisciplinary tracheostomy management worldwide.

## Author Contributions

**Charissa J. Zaga,** conceptualization, methodology, data curation, formal analysis, writing—original draft, writing—review and editing, project administration; **Carly E. Milliren,** conceptualization, methodology, data curation, formal analysis, writing original draft, writing—review and editing, project administration; **Brendan A. McGrath,** formal analysis, writing—review and editing, validation; **Christina J. Yang,** formal analysis, writing—review and editing, validation; **Bradley A. Schiff,** conceptualization, methodology, formal analysis, supervision, writing—review and editing; **Stephen J. Warrillow,** formal analysis, writing—review and editing, validation; **Jennifer K. Henningfeld,** formal analysis, writing—review and editing, validation; **Prudence A. Gregson,** formal analysis, writing—review and editing, validation; **Joshua R. Bedwell,** formal analysis, writing—review and editing, validation; **Karen M. Beaudet,** formal analysis, writing—review and editing, validation; **Michael J. Brenner,** conceptualization, methodology, data curation, formal analysis, writing—original draft, writing—review and editing, supervision, project administration; **Vinciya Pandian,** conceptualization, methodology, data curation, formal analysis, writing—original draft, writing—review and editing, supervision, project administration.

## Disclosures

### Competing interests

The authors declare no conflicts of interest.

### Funding source

R18 HS029124 and R01 NR017433 (Vinciya Pandian).

## Supporting information


**Supplemental Table S1**: STROBE checklist.

**Supplemental Table S2**: Country‐specific characteristics of sample. Demographics, clinical characteristics, comorbidities, hospital utilization, and survival are shown by country.

**Supplemental Table S3**: Country‐specific trends in decannulation over time. Trends in decannulation were assessed annually over 10 years.

**Supplemental Appendix:** Additional discussion of details regarding differences between countries and study limitations.

**Supplemental Figure S1**: Forest plot analysis of decannulation based on geography. Data show age, indications, admission type, comorbidities, and adverse events as predictors of decannulation.
