# Supplementary material for: Predictors of Decannulation Success in Tracheostomy: A 10‐Year Analysis of the Global Tracheostomy Collaborative Database
Source: Otolaryngol Head Neck Surg. 2025 Sep 2;173(5):1138–48. doi: 10.1002/ohn.70013 (PMC12574625; doi:10.1002/ohn.70013)
Supplement: Supplementary file 4 — Supplemental Appendix: Additional discussion of details regarding differences between countries and study limitations. [file OHN-173-1138-s003.docx]

**Supplemental Appendix 1.**

These disparities persist even after adjusting for patient factors, comorbidities, and hospital utilization, suggesting structural and protocol-based differences in tracheostomy care. The US cohort had a higher burden of comorbidities and lower rates of scheduled and surgical admissions, which likely contributed to lower decannulation rates, and a higher proportion of patients discharged to long-term care facilities.

Differences in primary admission diagnoses may further explain regional variations in decannulation outcomes and hospital utilization (p<0.001). The UK had the highest proportion of patients admitted with neurological conditions (38.9%), which are often associated with prolonged recovery and variability in decannulation timelines. The US had a greater proportion of respiratory failure cases (44.7%), a condition frequently linked to chronic ventilator dependence and lower decannulation success. In contrast, Australia had the largest proportion of head and neck pathology cases (29.1%), which often follow structured post-surgical decannulation pathways, potentially contributing to the country’s higher decannulation rates. These differences in case mix highlight the need to consider patient populations when interpreting decannulation success across healthcare systems.

Younger age, fewer comorbidities, and planned surgical admissions were associated with higher decannulation success, as expected. However, the observed geographic differences likely stem not only from patient factors but also from care processes, discharge planning, and structural variations in healthcare delivery, highlighting the importance of tailored, location-specific strategies to optimize outcomes. The geographic discrepancies observed in hospital length of stay, discharge destinations, and tracheostomy duration further highlight the impact of differing clinical workflows and post-discharge care models. For instance, the UK’s higher decannulation rates align with a more centralized tracheostomy care model, whereas the US model, with more fragmented post-acute care pathways, may result in extended tracheostomy dependence.

An unexpected finding was the higher rate of tracheostomy-related adverse events prior to decannulation among patients decannulated by discharge, since successful decannulation is generally regarded as a marker of favorable hospital course. One possible explanation is that unplanned decannulations predispose patients to adverse events, suggesting a need for vigilant monitoring and tailored intervention strategies. However, there was a higher incidence of failed decannulation attempts in successfully decannulated patients, reflecting the persistence sometimes required to achieve decannulation success. Bleeding and tube obstruction rates were not significantly different between decannulated and non-decannulated groups. This observation supports a potential reframing of repeated decannulation attempts as necessary steps toward recovery rather than as setbacks. In addition, it underscores the need for further study of decannulation protocols to identify which approaches allow for the greatest safety and efficiency.

Accidental decannulation was more common in patients who were ultimately successfully decannulated, suggesting a more nuanced role than just an adverse event. One explanation is that patients with fewer comorbidities and greater mobility may be more prone to unintentional decannulation. Alternatively, accidental decannulation may act as an unintentional "trial," revealing patient readiness for decannulation and prompting removal decisions. This challenges the traditional view of accidental decannulation and suggests a need for further research to assess whether such events could inform earlier decannulation trials rather than necessitating immediate reinsertion.

Additionally, this study focuses on decannulation during primary admission, excluding patients with long-term tracheostomies who may undergo decannulation in secondary admissions or outpatient settings. Future research should incorporate longitudinal tracking to capture extended tracheostomy care and refine standardized decannulation protocols. Lastly, while this dataset reflects practices up to June 2022, we were unable to analyze trends over time or assess the potential impact of COVID-19 on tracheostomy management. While we were unable to examine the direct influence of COVID-19, we acknowledge that the pandemic likely influenced ICU utilization, ventilator weaning, and discharge pathways. Future studies incorporating pandemic-era data are needed to explore long-term shifts in tracheostomy care and decannulation outcomes. Despite these limitations, this study provides one of the largest multinational analyses of adult tracheostomy decannulation and highlights opportunities for standardizing care to improve patient outcomes globally.
