# Supplementary figures and images for: Predictors of Decannulation Success in Tracheostomy: A 10‐Year Analysis of the Global Tracheostomy Collaborative Database
Source: Otolaryngol Head Neck Surg. 2025 Sep 2;173(5):1138–48. doi: 10.1002/ohn.70013 (PMC12574625; doi:10.1002/ohn.70013)

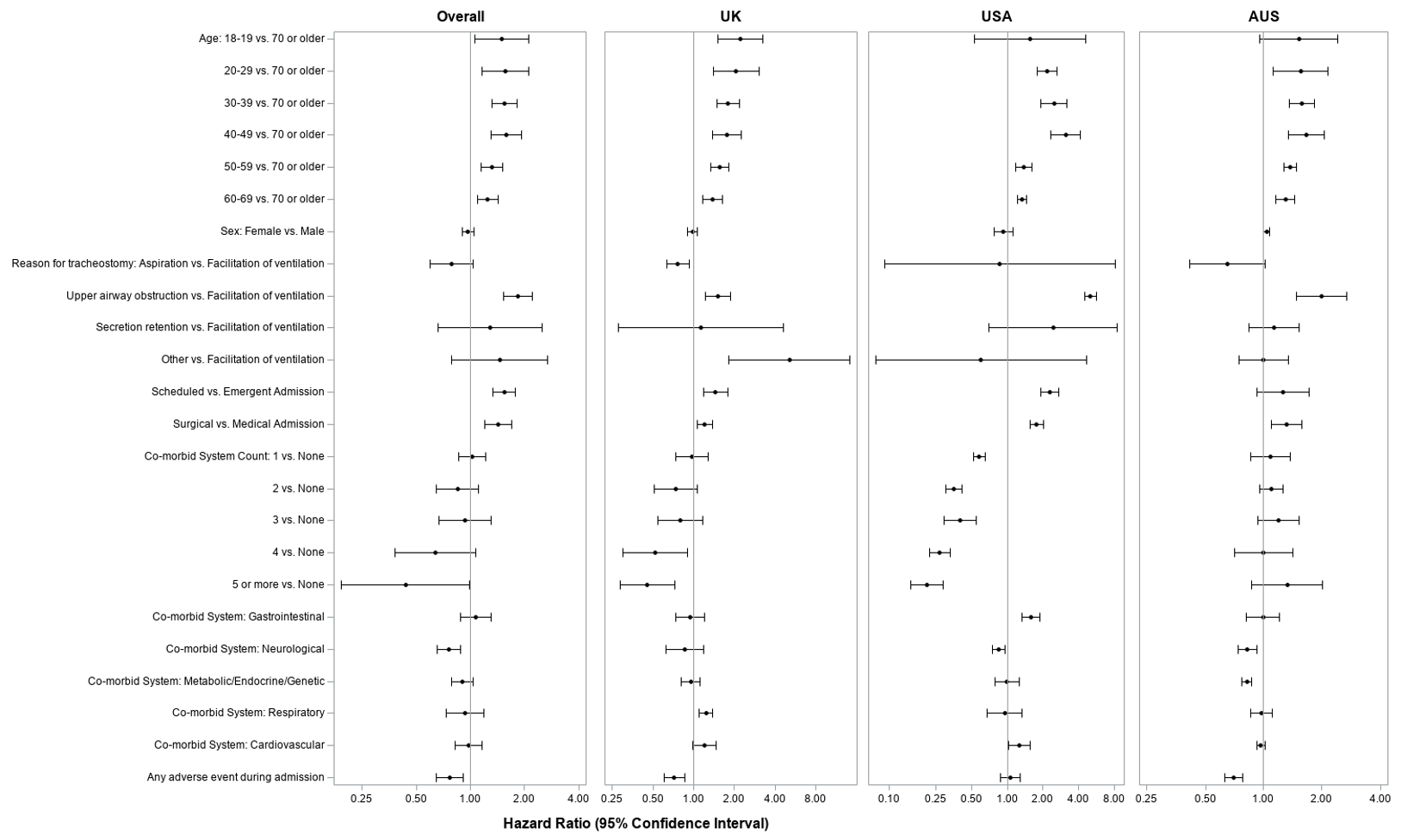

Supplement: Supplementary file 5 — Supplemental Figure S1: Forest plot analysis of decannulation based on geography. Data show age, indications, admission type, comorbidities, and adverse events as predictors of decannulation. [file OHN-173-1138-s004.png]
